# Effect of hydro-alcoholic extract of *Rosa damascena *on cardiovascular responses in normotensive rat

**Published:** 2015

**Authors:** Amir Baniasad, Abolfazl Khajavirad, Mahmoud Hosseini, Mohammad Naser Shafei, Saeed Aminzadah, Mahmoud Ghavi

**Affiliations:** 1 *Department of Physiology, School of Medicine, Mashhad University of Medical Sciences, Iran*; 2 *Neurocognitive Research Center, School of Medicine, Mashhad University of Medical Sciences, Iran*; 3 *Neurogenic Inflammation Research Centre, School of Medicine, Mashhad University of Medical Sciences, Mashhad, Iran*; 4 *Pharmacological Research Center of Medicinal Plants, School of Medicine, Mashhad University of Medical Sciences, Mashhad, Iran*

**Keywords:** *Rosa damascene*, *Blood pressure*, *Heart rate*, *Rat*

## Abstract

**Objective:**

*Rosa damascena *mill L. (*R. damascena*) is a well-known plant with fragrant effects. Several therapeutic effects of this plant on respiratory, gastrointestinal and nervous systems have been reported. It is also suggested to have beneficial effect on cardiovascular system especially blood pressure regulation. The present study was carried out to evaluate acute cardiovascular effect of hydro-alcoholic extract of *R. damascena*.

**Materials and Methods:**

Thirty-two male Wistar rats were randomly divided into four groups (n= 8 for each group). After anesthesia, a catheter was inserted into the femoral artery and blood pressure and heart rate (HR) were continuously recorded by a power lab system. Animals received three doses of hydro-alcoholic extract (250, 500, and 1000 mg/kg) via peritoneal (i.p). After 30 min, systolic blood pressure (SBP), mean arterial pressure (MAP) and HR were recorded and maximal changes were compared to control group.

**Results:**

Injection of all doses of the extract did not significantly change HR compare to control group. The SBP, dose dependently, was decreased by all doses of the extract and the maximal response was significant compared to saline group (p<0.01 to p<0.001). Different doses of the extract also dose-dependently decreased maximal changes of MAP responses compared to control group. The effect of higher doses of the extract on SBP and MAP was significant compared to lower doses (p<0.05 to p<0.01).

**Conclusion:**

This study provides evidence of a hypotensive effect of hydro-alcoholic extract of R. damascena with no significant effect on HR. Therefore, R. damascena is suggested to have beneficial effect to control blood pressure. However, it needs to be more investigated.

## Introduction

Medicinal plants have been used in various clinical conditions including cardiovascular regulation. One of these plants is *Rosa damascena mill L. (R. damascena)*. *R. damascena* is a member of the Rosaceae family that commonly known as damask rose (Rankouhi, 2009; Yassa et al., 2009 ▶). This plant has pink flowers and a perennial shrub that frequently used for fragrant effects and cultivated in Iran, Europe and Middle East countriesand Turkey (Ulusoy et al., 2009 ▶; Boskabady et al., 2011a ▶). *R. damascena* contains different components such as flavonoids, carboxylic acid, terpene, myrcene, geraniol (Babu et al., 2002 ▶), and vitamin C (Libster, 2002 ▶). In ancient medicine *R. damascena* has been used for different problems such as abdominal and chest pain (Wood et al., 1892 ▶), menstrual bleeding, digestive problems (Avesina, 1990 ▶), depression, grief, nervous stress and tension (Libster, 2002 ▶). Recent studies also have shown that *R. damascena* possesses multiple functions such as hypnotic, analgesic, bronchodilatory, antibacterial, laxative , prokinetic (Srinivasan et al., 2009 ▶; Shafei et al., 2010 ▶; Boskabady et al., 2011a ▶; Dolati et al., 2011b ▶; Kazerani & Behnam Rassouli, 2011 ▶), and anti-inflammatory effects (Avesina, 1990 ▶; Hajhashemi et al., 2010 ▶). Our previous studies also confirmed the antitussive, antidepressant, and excitatory effects on ileum contraction of this plant (Shafei et al., 2010 ▶; Dolati et al., 2011a; 2013 ▶). Few studies are available about the cardiovascular effect of *R. damascena*. It is previously reported that *R. damascena* has strengthening effect on heart (Wood et al., 1892 ▶). Boakabady et al. also reported that aqueous-ethanolic extract of *R. damascena* potentiates heart rate and contractility in isolated guinea-pig heart (Boskabady et al., 2013 ▶). In another study, adding ethanolic extract of *R. damascena* in diet for 45 days did not show any significant effect on cardiovascular system (Joukar et al., 2013 ▶). In addition, there are evidences that confirm effects of the plant on cardiovascular system. For example, Haze et al. reported that inhalation of rose oil caused decrease in sympathetic activity and adrenaline concentration (Haze et al., 2002 ▶). Another study evaluated the effect of rose oil on human autonomic parameters and showed that it could decrease systolic blood pressure (Hongratanaworakit, 2009 ▶). 

*R. damascena* also has active components such as flavonoids that those cardiovascular effects have been attributed to. In a previous study, Kwon et al. (2009) ▶ reported that flavonoids of buds of *R. damascena* contain cyanidin-3-O-beta-glucoside that significantly suppressed activity of angiotensin I converting enzyme (ACE) (Kwon et al., 2009 ▶), a key enzyme in formation of angiotensin II (Ang II) (Reid, 1992 ▶). Based on these evidences, we suggested that this plant has beneficial effect on cardiovascular parameters. Therefore, in this preliminary study, we examined the acute cardiovascular effect of the hydro-alcoholic extract of *R. damascena* in normotensive rats.

## Materials and methods

**Preparation of extract**

*R. damascena *was collected from Mashhad, Khorasan Razavi province, Iran, and identified by botanists in herbarium (No: 254-1804-01). We used maceration method in this study. Three hundred grams of dried flowers of *R. damascena* powdered then macerated in ethanol 70% for 72 hr. After that, the mixture was filtered. The solvent was evaporated by a rotary evaporator under reduced pressure at 50 ˚C. Concentrations of the extract were prepared by adding distilled water.

**Animals and groups**

Experiments were done using 42 male Wistar rats (220±20 g). Animals were housed at a temperature of 21-23 ˚C with free access to food and water. Rats were divided into four groups as follow (n = 8 in each group).

Group 1 (control): received saline.

Group 2 (rose 250): received 250 mg/kg of extract. 

Group 3 (rose 500: received 500 mg/kg extract. 

Group 4 (rose 1000): received 1000 mg/kg of extract. 

**Experimental procedure**

Animals were anesthetized with urethane (1.5 g/kg, i.p, with 0.7 g/kg as a supplementary dose). Temperature was kept at 37.5 ˚C with a heating lamp. A polyethylene catheter (PE-50) filled with heparinized saline was inserted in the femoral artery. The catheter was connected to a pressure transducer then cardiovascular parameters were continuously recorded by a power lab system (ID instrument, Australia (Shafei et al., 2013 ▶). To determine the cardiovascular effects of *R. damascena*, 30 min after injection (i.p) three doses(250, 500,1000 mg/kg) of hydro-alcoholic extract (Rakhshandah & Hosseini, 2006 ▶; Rakhshandah et al., 2010a ▶; Rakhshandah et al., 2010b ▶)., heart rate (HR), systolic blood pressure (SBP), and mean arterial pressure (MAP) were recorded (Shafei et al., 2013 ▶).

**Data analysis**

The results were expressed as mean ± SEM. The maximal changes of SBP (∆SBP), ∆MAP, and ∆HR were obtained and compared with the control group. Statistical comparisons between all groups were done using one-way ANOVA followed by Tukey’s test. *P* values less than 0.05 were considered for significant differences.

## Results

**Cardiovascular responses to injection of saline**

Injection of the saline (i.p., n = 8) had no significant effect on HR (baseline: 328.32±8.8 vs. saline: 318.62±7.9 beats/min), SBP (baseline: 84.4±4.2 vs. saline: 85.9±4.2 mm/Hg), and MAP; (baseline: 75.1±3.35 vs. saline: 76.1±3.1 mm/Hg).

**Effect of hydro-alcoholic extract of** ***R. damascena*** **on heart rate in anesthetized rat**

**Figure 1:**
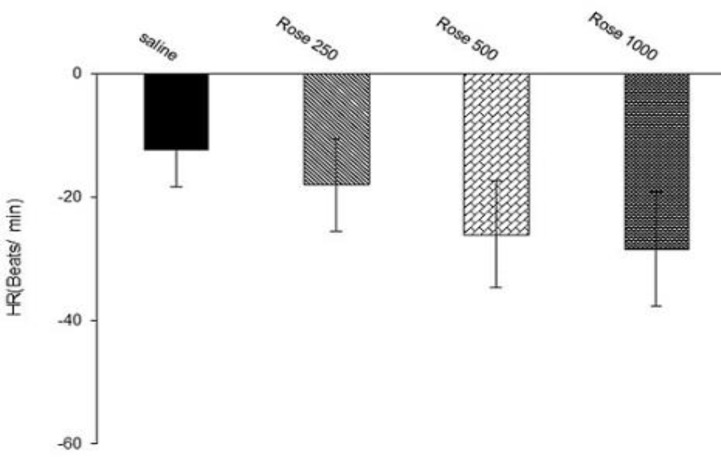
Effects of hydro-alcoholic extract of *R.* *damascena *on heart rate in anesthetized rats. Data were expressed as mean ± SEM. One-way ANOVA used for statistical analysis. (n=8).

To determine the effect of hydro-alcoholic extract on HR, three doses of extract were used. Administration of all doses of extract decreased HR, however, maximal changes of this effect was not significant compare to saline group (dose 250: ∆ -18.2±7.5, dose 500: ∆ -26.5.2±8.7, and dose 1000: ∆-28.4±9.3 beat/min vs. saline -12.4± 5.9 beat/min, n= 8 for each group, Figure 1)

**Effects of hydro-alcoholic extract of** ***R.damascena*** **on** **systolic blood pressure in normotensive rat**

**Figure 2:**
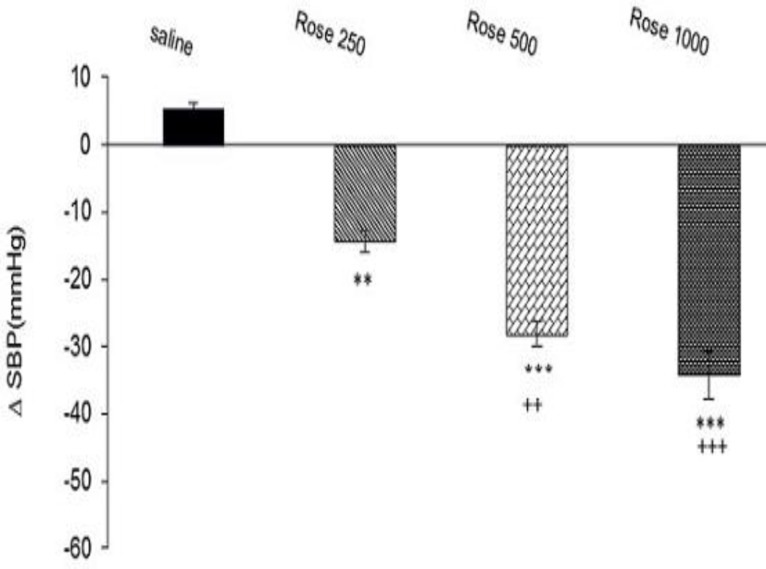
Effects of hydro-alcoholic extract of *R.* *damascena *on the SBP in anesthetized rats. Data were expressed as mean ± SEM. One-way ANOVA used for statistical analysis. (n=8).**: p<0.01, ***: p<0.001 *vs.* control, ++: p<0. 01, +++: p<0.001* vs.* dose 250

In this experiment, effect of three doses of extract on SBP was evaluated. As is been shown in Figure 2, SBP decreased by extract administration. Maximal SBP in dose 250 mg/kg (∆ -14.37±1.6 mm Hg, p<0.01), doses 500 mg/kg (∆ -28.12±1.8 mm Hg, p<0.001), and 1000 mg/kg (∆-34.14±4.5 mm Hg, p<0.001) was significant compare to saline group (∆ 5.4±0.8 mm Hg, n= 8 for each group, Figure 2). There were also significant differences between effect of two higher doses (500 and 1000 mg/kg) compare to low dose (p<0.05 to p<0.01, Figure 2).

**Effects of hydro-alcoholic extract of** ***R.damascena*** **on** **mean arterial pressure in normotensive rats**

**Figure 3:**
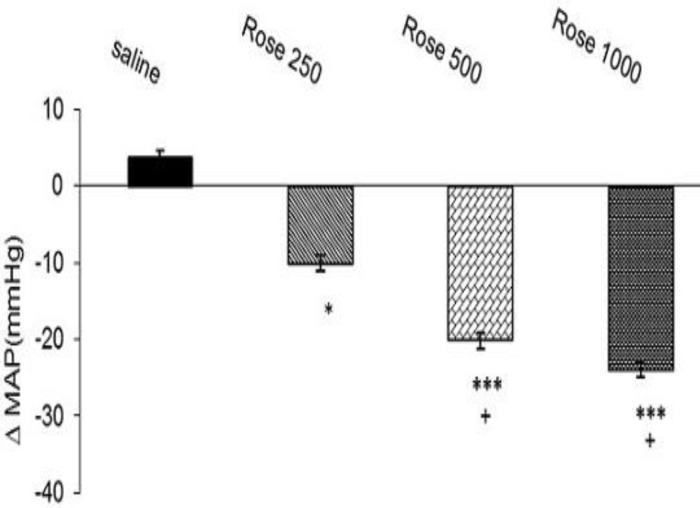
Effects of hydro-alcoholic extract of *R.* *damascena *on MAP in anesthetized rats. Data were expressed as mean ± SEM. One-way ANOVA used for statistical analysis. (n=8). **: p<0.01 and ***: p<0.001 vs. control, +: p<0.05 vs. dose 250

Effect of three doses of extract on MAP has been shown in Figure 3. As has been indicated, different doses of extract significantly decreased maximal ∆ MAP responses in doses of 250 mg (∆ -10.12±1.8 mm Hg, p<0. 05), 500 mg (∆ -20.24±2.34 mm Hg, p<0. 01), and 1000 mg (∆ -22.34±3.5 mm Hg, p<0. 001); n= 8 for each group) compared to saline (∆ 3.6±0.96 mm Hg). The results also indicated that effect of higher doses (500 and 1000 mg/kg) were significant compared to dose 250 mg/kg (p<.05, Figure 3).

## Discussion

The present study demonstrated that all doses of extract (250, 500, and 1000 mg/kg) significantly decreased SBP and MAP but had no significant effect on HR. 

The mechanism(s) of hypotensive effect of hydro-alcoholic extract of R. damascena cannot be concluded from present study but several suggested mechanisms are involved.

 In previous studies, the antispasmodic and relaxant effects of *R. damascena* have been indicated (Libster, 2002 ▶). Boskabady et al. also reported relaxing effect of this plant on guinea pig tracheal chains (Boskabady et al., 2006 ▶). With regard to relaxant effect of *R. damascena* on smooth muscles, the hypotensive response is probably mediated by vasodilator effect of extract.

A previous study showed that rose oil in human could decreased about 40% and 30% of sympathetic activity and adrenaline concentration, respectively (Haze et al., 2002 ▶) because blood pressure regulation is strongly affected by sympathetic system. It is possible that the hypotensive effect of this plant in the present study is mediated by inhibition of sympathetic activity. Mechanism of the effect of *R. damascena* on sympathetic activity is not yet clear but it is suggested that the effect is induced via mental and emotional conditions (Hongratanaworakit, 2009 ▶).

*R. damascena* contains several putative compounds including geraniol, myercene, terpene, and flavonoids (Loghmani-Khouzani et al., 2007; Boskabady et al., 2011a ▶). Flavonoids are polyphenolic compounds with several pharmacological properties including cardiovascular and antioxidant effects (Kris-Etherton & Keen, 2002 ▶). In a previous study, it was reported that cyanidin-3-O-β-glucoside isolated from *R. damascena* extract had inhibitory effect on ACE (Kwon et al., 2009 ▶). ACE is an important enzyme in converting angiotensin I into Ang II, a vasoconstrictor agent. Therefore, we suggest that hypotensive response of *R. damascena* is due to effect of this new flavonoid on production of Ang II. 

There are evidences that Ang II involves in the excitation of sympathetic system (Reid, 1992 ▶). Therefore, another possible mechanism of hypotensive effect of extract can be lower sympathetic activity induced by low concentration of Ang II. However, future studies are needed to clarify this opinion. 

Effect of R. damascena on blood pressure by cholinergic system is also suggested. It has been shown that acetylcholine (Ach) has inhibitory effects on cardiac and vascular smooth muscle (Adeneye et al., 2006 ▶). The vasorelaxant effects of acetylcholine are mostly mediated indirectly by stimulating the release of nitric oxide (NO) from the endothelium. NO also causes relaxation of smooth muscle by activation of guanylatcyclase (Furchgott & Zawadzki, 1980 ▶). Moreover, effect of *R. damascena* on cholinergic system in gastrointestinal tract is reported (Dolati et al., 2013 ▶). It is possible that this plant by its effect on cholinergic system decreased blood pressure.

In another study, Boskabady et al. showed that *R. damascena* increased HR and contractility in isolated heart of guinea pigs (Boskabady et al., 2011b ▶; Boskabady et al., 2013 ▶). It has also been reported that this effect is mediated by beta adrenergic receptors (Boskabady et al., 2013 ▶). Our result in partly is opposite to this result. This study evaluated effect of R. damascena in vitro (isolated heart), however, we studied in vivo (anesthetized rats). In addition, it is possible that the effect of R. damascena on adrenergic system is mediated by both alpha and beta receptors. With regard to the difference in type of adrenergic receptors of heart (beta) and vessels (alpha), we suggest that the effect of extract on heart and vessels are different. However, future studies are needed to clarify the effects of extract on adrenoceptors. 

 Antioxidant and anti-inflammation effects of R. damascena have also been reported (Hajhashemi et al., 2010 ▶; Boskabady et al., 2011a ▶). Because oxidative stress and inflammation play important roles in cardiovascular regulation, hypotensive effect of this plant maybe mediated by anti-oxidative and anti-inflammation properties of *R. damascena.*

In summary, this study provides evidence of hypotensive effect of hydro-alcoholic extract of R. damascena. However, further studies are needed to clarify the effective substances of this plant and those mechanisms involving cardiovascular system.
